# The cobas^®^ HCV GT is a new tool that accurately identifies Hepatitis C virus genotypes for clinical practice

**DOI:** 10.1371/journal.pone.0175564

**Published:** 2017-04-14

**Authors:** J. A. Fernández-Caballero, M. Alvarez, N. Chueca, A. B. Pérez, F. García

**Affiliations:** Servicio de Microbiología, Complejo Hospitalario Universitario Granada-Hospital PTS, Instituto de Investigación Biosanitaria IBS, Granada, Spain; National Taiwan University Hospital, TAIWAN

## Abstract

**Objective:**

We aimed to evaluate the correct assignment of HCV genotype/subtypes 1a and 1b by cobas^®^ HCV genotyping (GT) assay (Roche Molecular Diagnostics) compared with nonstructural protein 5B (NS5B) sequencing.

**Patients and methods:**

Clinical samples from 153 patients submitted for HCV genotyping were studied. After genotyping with the cobas^®^ HCV GT, sequencing of a 387 bp fragment in the NS5B gene and phylogenetic analysis was employed to compare genotyping results. Major discrepancies were defined as differences in the assigned genotype by cobas^®^ HCV GT and NS5B sequencing (including genotype 1 subtypes 1a and 1b misclassification).

**Results:**

Overall agreement between the cobas^®^ HCV GT and NS5B sequencing was 98%; all the 1a, 1b, 2, 3 and 4 genotypes identified by cobas^®^ HCV GT were concordant with NS5B sequencing. Three samples tested “indetermined” by cobas^®^ HCV GT assay and were genotyped as 1a, 3a, and 4d by NS5B sequencing.

**Conclussion:**

These results indicate that the cobas^®^ HCV GT assay correctly identifies HCV genotypes, and points out the importance of additional methods based on DNA sequencing for resolving indeterminate results.

## Introduction

Hepatitis C virus genotyping is still a key component for proper management of chronic hepatitis C (CHC) [1]. Allthough the number of pangenotypic direct-acting antivirals (DAAs) licensed for treatment of CHC is continuously increasing, determination of the HCV genotype (GT) including the HCV GT 1 subtypes 1a and 1b is still recommended before starting antiviral therapy. The HCV GT has an influence on the combination of the direct antiviral agent (DAA), the combination of DAAs, the need to add ribavirin to the regimen, and treatment duration [1–3]. Genotyping may also give insight for molecular epidemiology studies [4], and may also be important for the interpretation of resistance-associated substitutions (RAS) [5].

HCV is classified into seven genotypes (1 to 7) and into 67 confirmed and some provisional subtypes [6]. Commercial HCV genotyping assays target different HCV genomic regions (e.g. 5’-UTR, Core, NS5B) and are currently based on reverse hybridization [VERSANT HCV Genotype 2.0 assay (Siemens)] or real time PCR assays [Real-Time HCV genotype II (Abbott)]. DNA sequencing of the nonstructural protein 5B (NS5B) is an alternative to commercial HCV genotyping [7].

The cobas^®^ HCV GT (Roche Molecular Diagnostics, Pleasanton, CA) is a new automated commercial assay that uses real time PCR for identification of genotypes 1 to 6 and subtypes 1a and 1b. Here we describe the performance of this test compared to DNA sequencing followed by phylogenetic analysis of the NS5B region.

## Materials and methods

As an extension of the GEHEP-007 study [8], we have conducted a prospective study, including all consecutive clinical samples submitted from February 2016 to June 2016 for HCV genotyping to our laboratory. All samples were tested employing the cobas^®^ HCV GT according to the manufacturer´s package insert. For NS5B sequencing, the extracted RNA eluate was transcribed to cDNA using RevertAid H Minus First Strand cDNA Synthesis kit (Thermo Scientific) and an internal fragment of 371 bp in the NS5B gene (nucleotide positions 8325 to 8695) was amplified and used for DNA sequencing; HCV genotype was assigned using geno2pheno HCV (http://hcv.geno2pheno.org/index.php), MoleBlast (http://hcv.lanl.gov/content/sequence/BASIC_BLAST/basic_blast.html) and phylogenetic trees were reconstructed by maximum likelihood (ML) with PhyML 3.0 using the general time reversible plus proportion of invariable sites, plus gamma distribution and a BIONJ starting tree. The ethics committee of the San Cecilio Hospital approved the study, and no consent information was required as patient information was anonymised and de-identified prior to analyses.

For data analysis, discordant results were defined as differences in the assigned genotype by cobas^®^ HCV GT and NS5B sequencing (including genotypes 1a and 1b misclassification).

## Results

Samples from 153 patients were included in the study. Patients were mostly Spanish (97.4%), males (69.9%), with a median age of 52.5 (IQR: 47–65), a median HCV viral load of 5.9 log IU/ml (IQR: log 4.9–6.5). The cobas^®^ HCV GT identified 55 samples as GT 1b (35.9%), 54 as GT 1a (35.3%), one as GT 2 (0.7%), 22 as GT 3 (14.4%) and 18 as GT 4 (11.8%). Samples from three patients (2%) could not be genotyped by the cobas^®^ HCV GT as they were called as indeterminate by this test (defined by the manufacturer as detection of HCV control but no genotype or subtype identified). The overall concordance with respect to the reference method was 98%; all the 1a, 1b, 2, 3 and 4 genotypes identified by cobas^®^ HCV GT were concordant with NS5B sequencing, while the three indeterminates by cobas^®^ HCV GT were genotyped as 1a, 3a, and 4d by NS5B sequencing. In addition, NS5B sequencing assigned the HCV subtype for all the 2, 3 and 4 genotypes identified by cobas^®^ HCV GT. These resuts are shown in Table 1.

**Table 1:** HCV genotyping and subtyping results using cobas^®^ HCV GT and NS5B sequencing.

cobas^®^ HCV GT (n)	NS5B DNA Sequencing
1a (54)	1a (54)
1b (55)	1b (55)
2 (1)	2c (1)
3 (22)	3a (22)
4 (18)	4a (6); 4d (12)
Indeterminate (3)	1a (1); 3a (1); 4d (1)

## Discussion

Treatment success and patient cure with DAA based regimens not including a combination of pangenotypic drugs depends on a correct identification of HCV genotype. Until all oral pangenotypic regimens become available, treatment guidelines [1, 2] stress on the accurate estimation of HCV genotype for the selection of the treatment regimen, duration and the need for using ribavirin.

In this study we compared the cobas^®^ HCV GT and DNA sequencing of NS5B. In contrast to other commercially available tests fot HCV genotyping, this test is based on real-time PCR of the 5’UTR region of HCV for the identification of HCV GT 2,3 and 6, real-time PCR of the core region for the identification of HCV GT 1,4 & 5, and real-time PCR of the NS5B region for the identification of HCV subtypes 1a and 1b. Abbott Real Time HCV Genotype II test has a similar design but does not include the analysis of the core region and Versant HCV Genotype 2.0 assay (LIPA) is based on reverse hybridization of PCR amplicons of the 5’UTR and core regions, but does not include NS5B for 1a/1b differentiation.

In our previous study [8], we compared Abbott Real Time HCV Genotype II assay, Versant HCV Genotype 2.0 assay (LIPA) and Trugene HCV Genotyping Kit, to NS5B sequencing, and we described a high concordance of the Abbott Real Time HCV Genotype II assay. Here, we also describe a high concordance of cobas^®^ HCV GT with NS5B sequencing; in fact, all the samples that could be genotyped with the commercial assay were concordant with the reference test. However, 2% of the samples were called indetermined by cobas^®^ HCV GT, and for these samples DNA sequencing of NS5B did ascribe the HCV genotype. Some other studies have also reported different rates of indeterminate calls for the Abbott Real Time HCV Genotype II assay (reported the same problem in 4% of their 343 patients [9]; 5.4% [10]; 6.1% [11]; 9–10% [12]), and did also need to refer to NS5B sequencing for a correct assignment of HCV GT. More recently, Stelzl et al [13] also reported similar findings regarding indeterminates (3.8%).

In our study, the main limitation was the sample size for HCV GT 2 (0.6%), which may not be representative of the true prevalence in Spain or Europe of this genotype; in a recent large Spanish genotypic survey including 48.947 patients [5], HCV GT 2 represents 2.8% of the population. In order to avoid this limitation we searched our database for stored GT 2 samples by NS5B sequencing and re-runed them with the cobas^®^ HCV GT assay. All the seven HCV GT2 samples tested were concordant.

In summary, we found a high concordance of cobas^®^ HCV GT with the reference test for HCV genotyping. For a low number of samples, cobas^®^ HCV GT could not assign HCV genotype. Access to reference tests for HCV genotyping in clinical microbiology laboratories, such as NS5B Sanger sequencing, should take part as one of the efforts to achieve higher sustained viral response rates in HCV treatment, and to contribute to the cure and eradication of Hepatitis C virus.
